# Centile values for serum lipids and blood pressure for Asian Indian adolescents

**DOI:** 10.1186/1476-511X-4-20

**Published:** 2005-09-29

**Authors:** Malini Madhavan, Ravindra M Pandey, Anoop Misra, Naval K Vikram, Vibha Dhingra, Kalpana Luthra, Jasjeet S Wasir

**Affiliations:** 1 Department of Medicine, All India Institute of Medical Sciences New Delhi-110029, India; 2 Department of Biostatistics, All India Institute of Medical Sciences New Delhi-110029, India; 3 Department of Biochemistry, All India Institute of Medical Sciences New Delhi-110029, India

**Keywords:** Asian Indians, adolescents, centiles, lipids, blood pressure.

## Abstract

**Background:**

Reference data for plasma lipids and blood pressure are not available for Asian Indian adolescents. This study aimed to develop representative age- and sex- specific percentile reference data for serum lipids [total cholesterol (TC), triglycerides (TG), low density lipoprotein cholesterol (LDL-C), high density lipoprotein cholesterol (HDL-C), non-HDL cholesterol] and blood pressure for urban Asian Indian adolescents aged 14–18 years. The sample consisted of 680 boys and 521 girls aged 14–18 years from the cross-sectional population survey, *E*pidemiological *S*tudy of *A*dolescents and *Y*oung Adults (ESAY) for whom the data for serum lipid levels and blood pressure were recorded. Smoothed age- and sex- specific 5^th^, 10^th^, 25^th^, 50^th^, 75^th^, 85^th^, 90^th ^and 95^th ^percentiles where derived using LMS regression.

**Results:**

Percentile-based reference data for serum lipids and blood pressure are presented for adolescent Asian Indian boys and girls for the first time. Asian Indian adolescents had lower levels of serum TC, LDL-C and HDL-C and higher TG than their counterparts in the USA. Interesting trends in TC and HDL-C levels where observed, which might reflect changes in dietary pattern and physical activity in this age group in India.

**Conclusion:**

These reference data could be used to identify adolescents with an elevated risk of developing dyslipidemia, hypertension and cardiovascular disorders, to plan and implement preventive policies, and to study temporal trends.

## Introduction

Atherosclerosis begins in childhood and progresses to coronary heart disease (CHD) in adults [1]. Aortic fatty streaks and fibrous plaques occur in children and adolescents [2-4]. The risk factors for atherosclerosis, such as dyslipidemia, hypertension, and insulin resistance may arise in children and adolescents, and contribute significantly to the acceleration of atherosclerosis [1].

Tracking of total cholesterol (TC) and low-density lipoprotein cholesterol (LDL-C) levels from childhood to adults is well known. The National Cholesterol Education Program, Adult Treatment Panel III (NCEP, ATP III) identified LDL-C as the primary target for cholesterol-lowering therapy [5]. Elevated triglycerides (TG) and low levels of high-density lipoprotein cholesterol (HDL-C) are markers of atherogenic dyslipidemia [6-8]. Specifically, low levels of HDL-C is an independent risk factor for CHD [9]. Non-HDL cholesterol also shows strong correlation with CHD mortality [10] and has been recommended as a secondary target of therapy [5].

CHD is increasingly becoming a leading cause of death in India and other developing Asian countries. South Asians have premature and severe CHD as compared to white Caucasians [11]. Important contributory factors for CHD in South Asians are insulin resistance and resultant dyslipidemia [12]. We have recently reported that insulin resistance and low levels of HDL-C are common in Asian Indian adolescents, portending high risk for development of CHD in adults [13,14].

No representative percentiles reference data for plasma lipids and blood pressure in Asian Indian adolescents are currently available. These data are required for proper diagnosis and prevention of dyslipidemia, hypertension, and CHD. The purpose of this study was to develop representative age- and sex- specific percentile reference data for serum lipids (TC, LDL-C, HDL-C, TG, non-HDL cholesterol), and blood pressure [systolic blood pressure (SBP) and diastolic blood pressure (DBP)] for urban Asian Indian adolescents aged 14–18 years (y).

## Methods

### Subjects

The data were taken from an epidemiological study involving adolescents and young adults (aged 14–25 y) from schools and colleges located in southwest area of New Delhi. Multistage cluster sampling based on modified World Health Organization Expanded Program of Immunization Sampling Plan was used to collect a representative sample of adolescents and young adults [15]. First, two separate lists, one containing the names of schools and the other containing the names of colleges located in the defined area were prepared. A 'cluster' was defined as a school or a college. A total of 40 clusters were randomly selected from the two lists. The number of schools and colleges was determined based on the proportional allocation to ensure the representativeness of the sample with respect to clusters and socioeconomic strata. For both schools and colleges, a 'section' was considered as the primary sampling unit at the second stage of sampling. Subsequently from each of the schools/colleges, two to four sections were selected depending upon the number of the students in the section. All the students in the selected section were included in the study. Informed consent was obtained from the parents of the selected children. The institutional ethics committee approved the study. The data set consists of 680 boys and 521 girls aged 14 to 18 y. The sample characteristics are presented in table 1.

**Table 1:** Mean (SD) of lipid levels and blood pressure in urban Asian Indian adolescents 14–18 y of age

*Age (y)*	*n*		*TC (mg/dl)*		*LDL-C(mg/dl)*		*TG (mg/dl)*		*HDL-C (mg/dl)*		*SBP (mmHg)*		*DBP(mmHg)*	
	
	Boys	Girls	Boys	Girls	Boys	Girls	Boys	Girls	Boys	Girls	Boys	Girls	Boys	Girls
14	58	65	148.9 (21.0)	148.4 (26.0)	83.8 (20.3)	80.7 (26.5)	92.4 (37.8)	95.8 (28.5)	46.5 (8.5)	48.2 (10.3)	113.9 (10.3)	111.6 (9.6)	72.6 (7.6)	72.0 (7.0)
15	134	109	148.9 (27.3)	155.4 (23.6)	84.2 (27.3)	87.8 (23.9)	86.7 (30.5)	92.0 (28.0)	47.4 (7.9)	48.3 (8.3)	112.7 (9.8)	111.7 (8.9)	73.4 (7.4)	73.2 (7.0)
16	218	155	149.3 (24.0)	153.8 (21.4)	82.1 (25.8)	86.4 (23.2)	89.0 (29.7)	96.7 (28.0)	47.9 (6.1)	48.0 (8.5)	114.1 (9.5)	111.6 (9.3)	73.9 (7.0)	72.8 (6.6)
17	182	84	142.3 (27.9)	152.3 (21.4)	77.8 (27.9)	83.2 (24.7)	86.3 (32.1)	92.7 (29.0)	48.2 (6.9)	49.5 (7.8)	115.6 (9.2)	109.7 (9.9)	75.6 (6.4)	71.3 (7.3)
18	88	108	136.9 (24.1)	158.1 (23.8)	71.7 (22.1)	84.3 (24.7)	89.1 (34.0)	92.3 (28.9)	47.7 (7.0)	55.6 (7.6)	116.0 (9.0)	109.8 (8.2)	75.5 (6.7)	72.5 (7.2)

TC, Total cholesterol; LDL-C, Low-density lipoprotein cholesterol; TG, Triglycerides; HDL-C, High-density lipoprotein cholesterol; SBP, Systolic blood pressure; DBP, Diastolic blood pressure.

For conversion to mmol/L for TC, LDL-C and HDL-C, multiply by 0.02586

For conversion to mmol/L for TG, multiply by 0.01129

### Lipoproteins

For estimation of lipoproteins, blood samples were obtained after an overnight fast of 10 hours. Levels of TC, TG, and HDL-C were estimated using commercially available kits (Randox Laboratory, San Francisco, CA, USA) on a semi-automated analyzer (das srl, palombara, Sabina, Italy) as described previously [13,16]. Value of LDL-C was calculated according to the Friedewald's equation [17]. The estimation of all lipids was rigorously quality controlled by a consultant biochemist (KL), and frequently checked with values of another reference laboratory. Inter-assay and intra-assay variability of estimations were kept at less than 5%.

### Blood Pressure

Blood pressure was measured by a standard mercury sphygmomanometer (Industrial Electronic and Allied Products, Pune, India), after the subject had rested for 5 min in the sitting position, using the appropriate cuff size. Phase 5 Korotkoff sounds were taken for diastolic blood pressure categorization. In case of an abnormal blood pressure recording, another reading was obtained after 5 min rest and the mean of the two values was taken for the final record. The same physician measured the blood pressure using the same instrument for all the subjects. The mercury sphygmomanometer was periodically validated against a Hawksley Random Zero Sphygmomanometer (Hawksley, Lancing, Sussex, UK).

### Statistical methods

The data were first examined for outliers. The LMS method was used to obtain smoothed centile curves for each of the anthropometric variables. The need for centile curve arises when the measurement is strongly dependent on some covariate, often age, so that the reference range changes with the covariate. The LMS method uses Box-Cox power transformation, which deals with the skewness present in the distribution of the anthropometric measurement and provides a way to normalize the measurement. The final centile curves are the result of smoothing three-age specific curves called L (lambda), M (mu) and S (sigma). The M and S curves correspond to the median and coefficient of variation of the measurement at each age whereas the L curve allows for the substantial age dependent skewness in the distribution of the measurement. The points on each centile curves are defined by the following formula:

M(1+LSz)^1/L^,

where L, M and S are the values of the fitted curves at each age, and z denotes the z score, i.e. the standard score with mean 0 and a standard deviation of 1, for the required centile, for example z = 1.645 for the 95^th ^centile. The main assumption underlying the LMS method is that after Box-Cox power transformation the data at each age are normally distributed.

Descriptive statistics were computed using STATA 8.0 intercooled version (STATA Corporation, College Station Road, Houston, Texas) and the LMS regressions were performed using LMS Pro software (The Institute of Child Health, London).

## Results

Smoothed age- and sex- specific cut-offs of serum TC, LDL-C, HDL-C, TG, non-HDL-C, SBP and DBP at the 5^th^, 10^th^, 25^th^, 50^th^, 75^th^, 85^th^, 90^th^, and 95^th ^percentiles are presented in tables 2, 3, 4, 5, 6, 7, 8. Figures 1, 2, 3, 4, 5, 6, 7 present the smoothed percentile curves graphically for boys and figures 8, 9, 10, 11, 12, 13, 14 present the smoothed percentile curves graphically for girls.

**Table 2:** Smoothed age- and sex- specific serum total cholesterol (mmol/l) percentile values for urban Asian Indian adolescents 14–18 y of age.

	*Age*	*5*^*th*^	*10*^*th*^	*25*^*th*^	*50*^*th*^	*75*^*th*^	*85*^*th*^	*90*^*th*^	*95*^*th*^
Boys									
	14	2.98	3.15	3.45	3.82	4.22	4.45	4.62	4.88
	15	2.87	3.06	3.40	3.81	4.25	4.51	4.69	4.97
	16	2.84	3.04	3.39	3.82	4.29	4.56	4.75	5.04
	17	2.68	2.86	3.19	3.61	4.08	4.36	4.56	4.88
	18	2.60	2.77	3.08	3.47	3.94	4.22	4.43	4.76
Girls									
	14	2.93	3.09	3.38	3.76	4.20	4.47	4.67	4.99
	15	3.09	3.27	3.60	3.99	4.40	4.64	4.80	5.05
	16	3.07	3.26	3.59	3.97	4.35	4.56	4.70	4.91
	17	3.05	3.23	3.56	3.93	4.31	4.51	4.66	4.87
	18	3.16	3.35	3.67	4.05	4.47	4.71	4.88	5.13

**Table 3:** Smoothed age- and sex- specific percentile values for low-density lipoprotein cholesterol (mmol/l) for urban Asian Indian adolescents 14–18 y of age.

	*Age*	*5*^*th*^	*10*^*th*^	*25*^*th*^	*50*^*th*^	*75*^*th*^	*85*^*th*^	*90*^*th*^	*95*^*th*^
Boys									
	14	1.24	1.43	1.76	2.14	2.52	2.73	2.88	3.09
	15	1.15	1.37	1.74	2.18	2.63	2.88	3.06	3.31
	16	1.10	1.29	1.65	2.09	2.57	2.84	3.03	3.32
	17	1.04	1.21	1.53	1.94	2.42	2.70	2.91	3.23
	18	0.98	1.13	1.41	1.78	2.22	2.49	2.69	3.00
Girls									
	14	1.12	1.30	1.64	2.05	2.51	2.76	2.94	3.22
	15	1.24	1.46	1.82	2.24	2.68	2.92	3.09	3.34
	16	1.23	1.45	1.81	2.22	2.64	2.86	3.01	3.24
	17	1.18	1.39	1.75	2.16	2.57	2.80	2.95	3.19
	18	1.22	1.40	1.73	2.14	2.58	2.84	3.01	3.29

**Table 4:** Smoothed age- and sex- specific percentile values for high-density lipoprotein cholesterol (mmol/l) for urban Asian Indian adolescents 14–18 y of age.

	*Age*	*5*^*th*^	*10*^*th*^	*25*^*th*^	*50*^*th*^	*75*^*th*^	*85*^*th*^	*90*^*th*^	*95*^*th*^
Boys									
	14	0.86	0.93	1.05	1.20	1.35	1.43	1.48	1.57
	15	0.91	0.98	1.10	1.23	1.36	1.43	1.48	1.55
	16	0.96	1.02	1.12	1.24	1.35	1.41	1.46	1.52
	17	0.98	1.04	1.13	1.24	1.36	1.42	1.47	1.54
	18	0.98	1.03	1.11	1.21	1.33	1.41	1.47	1.56
Girls									
	14	0.86	0.93	1.07	1.24	1.41	1.51	1.58	1.68
	15	0.88	0.96	1.09	1.24	1.40	1.48	1.54	1.63
	16	0.90	0.97	1.10	1.24	1.38	1.46	1.52	1.60
	17	0.96	1.03	1.14	1.28	1.41	1.49	1.54	1.62
	18	1.12	1.19	1.30	1.43	1.57	1.64	1.70	1.77

**Table 5:** Smoothed age- and sex- specific percentile values for serum triglycerides (mmol/l) for urban Asian Indian adolescents 14–18 y of age.

	*Age(y)*	*5*^*th*^	*10*^*th*^	*25*^*th*^	*50*^*th*^	*75*^*th*^	*85*^*th*^	*90*^*th*^	*95*^*th*^
Boys									
	14	0.55	0.62	0.75	0.95	1.22	1.40	1.54	1.79
	15	0.53	0.60	0.73	0.92	1.16	1.32	1.44	1.63
	16	0.54	0.61	0.76	0.95	1.20	1.35	1.46	1.65
	17	0.51	0.59	0.73	0.92	1.16	1.31	1.43	1.61
	18	0.54	0.60	0.74	0.94	1.19	1.36	1.49	1.71
Girls									
	14	0.62	0.70	0.85	1.04	1.27	1.41	1.51	1.66
	15	0.60	0.68	0.82	1.00	1.22	1.35	1.45	1.60
	16	0.64	0.71	0.86	1.05	1.28	1.42	1.52	1.68
	17	0.60	0.67	0.82	1.01	1.23	1.37	1.47	1.62
	18	0.58	0.66	0.81	1.00	1.23	1.37	1.47	1.64

**Table 6:** Smoothed age- and sex- specific percentile values for non-HDL cholesterol (mmol/l) for urban Asian Indian adolescents 14–18 y of age.

	*Age(y)*	*5*^*th*^	*10*^*th*^	*25*^*th*^	*50*^*th*^	*75*^*th*^	*85*^*th*^	*90*^*th*^	*95*^*th*^
Boys									
	14	1.71	1.91	2.24	2.62	3.01	3.23	3.38	3.60
	15	1.60	1.81	2.19	2.63	3.08	3.34	3.52	3.78
	16	1.56	1.76	2.13	2.57	3.05	3.33	3.52	3.82
	17	1.46	1.63	1.95	2.36	2.83	3.12	3.32	3.64
	18	1.42	1.56	1.85	2.22	2.68	2.97	3.19	3.53
Girls									
	14	1.66	1.84	2.17	2.56	2.98	3.23	3.40	3.65
	15	1.79	1.99	2.35	2.75	3.17	3.40	3.56	3.80
	16	1.77	1.98	2.33	2.73	3.13	3.35	3.50	3.72
	17	1.70	1.90	2.25	2.64	3.06	3.28	3.44	3.67
	18	1.67	1.86	2.20	2.61	3.06	3.32	3.50	3.78

**Table 7:** Smoothed age- and sex- specific percentile values for systolic blood pressure (mm Hg) for urban Asian Indian adolescents 14–18 y of age.

	*Age(y)*	*5*^*th*^	*10*^*th*^	*25*^*th*^	*50*^*th*^	*75*^*th*^	*85*^*th*^	*90*^*th*^	*95*^*th*^
Boys									
	14	99	103	108	115	121	125	127	131
	15	98	101	107	113	119	123	125	128
	16	98	102	108	114	120	123	125	129
	17	100	103	109	115	121	125	127	130
	18	102	105	110	116	122	125	128	131
Girls									
	14	96	100	105	112	118	121	123	127
	15	97	100	105	111	118	121	123	127
	16	97	100	105	111	118	121	124	127
	17	95	98	103	109	116	119	121	125
	18	96	99	104	110	116	119	121	124

**Table 8:** Smoothed age- and sex- specific percentile values for diastolic blood pressure (mm Hg) for urban Asian Indian adolescents 14–18 y of age.

	*Age(y)*	*5*^*th*^	*10*^*th*^	*25*^*th*^	*50*^*th*^	*75*^*th*^	*85*^*th*^	*90*^*th*^	*95*^*th*^
Boys									
	14	61	64	68	73	78	81	82	85
	15	62	64	69	74	79	81	83	85
	16	62	65	69	74	78	81	82	85
	17	64	67	71	75	80	82	84	86
	18	64	67	71	76	80	82	84	86
Girls									
	14	60	63	68	72	77	79	81	83
	15	62	64	69	73	78	80	82	84
	16	62	64	68	73	77	80	81	84
	17	60	63	67	71	76	78	80	83
	18	61	63	68	72	77	80	82	85

**Figure 1:**
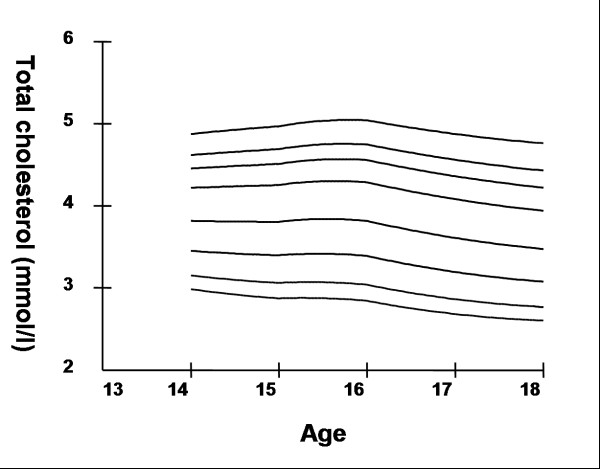
Smoothed percentile curves for the 5^th^, 10^th^, 25^th^, 50^th^, 75^th^, 85^th^, 90^th ^and 95^th ^percentiles of total cholesterol for urban boys 14–18 years of age.

**Figure 2:**
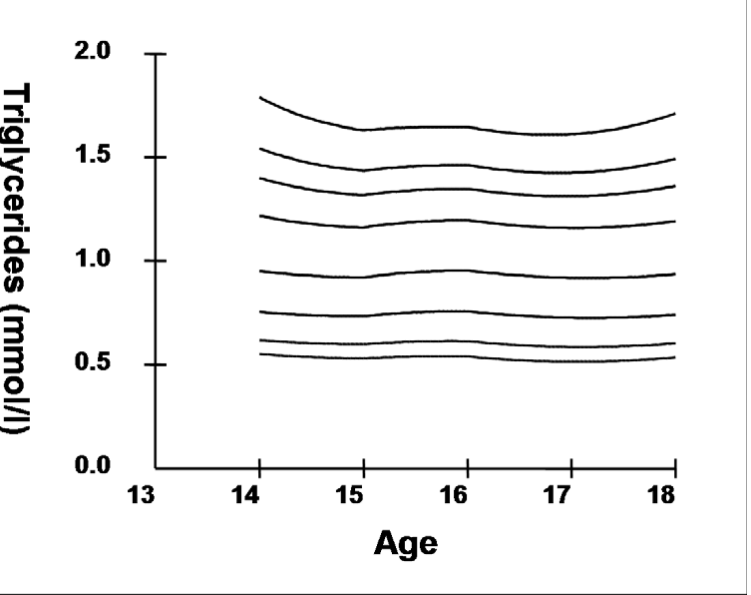
Smoothed percentile curves for the 5^th^, 10^th^, 25^th^, 50^th^, 75^th^, 85^th^, 90^th ^and 95^th ^percentiles of serum triglycerides for urban boys 14–18 years of age.

**Figure 3:**
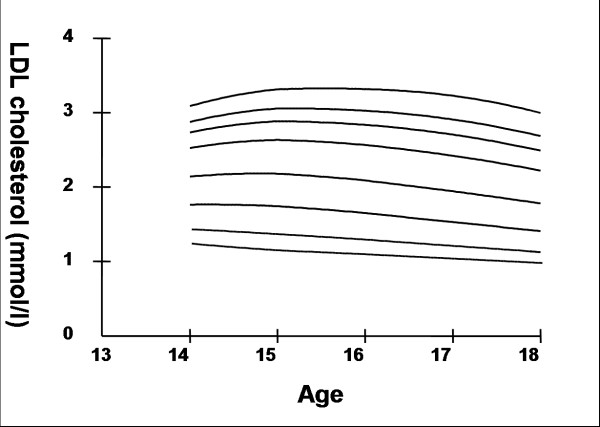
Smoothed percentile curves for the 5^th^, 10^th^, 25^th^, 50^th^, 75^th^, 85^th^, 90^th ^and 95^th ^percentiles of low-density lipoprotein cholesterol for urban boys 14–18 years of age.

**Figure 4:**
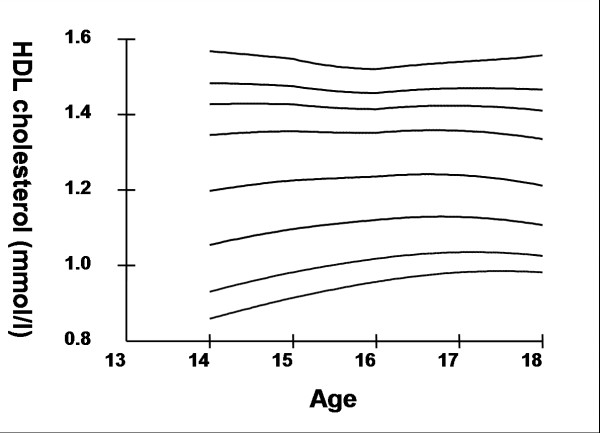
Smoothed percentile curves for the 5^th^, 10^th^, 25^th^, 50^th^, 75^th^, 85^th^, 90^th ^and 95^th ^percentiles of high-density lipoprotein cholesterol for urban boys 14–18 years of age.

**Figure 5:**
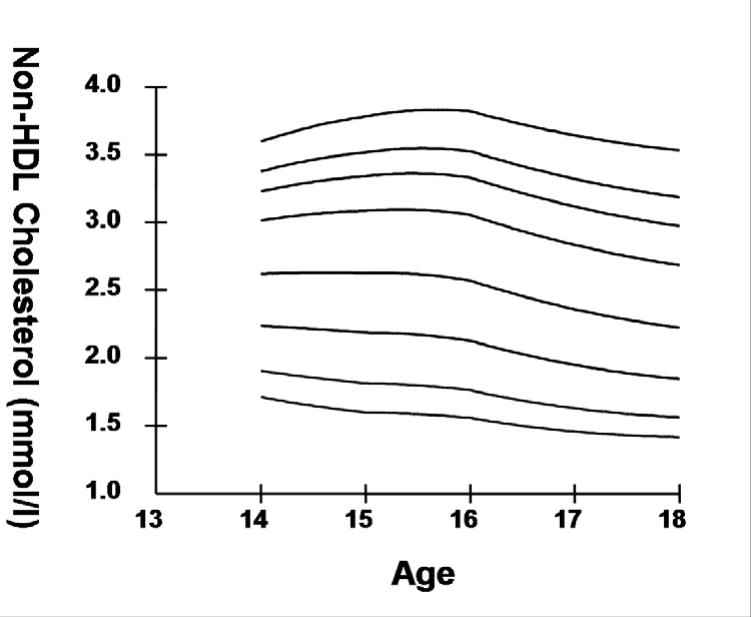
Smoothed percentile curves for the 5^th^, 10^th^, 25^th^, 50^th^, 75^th^, 85^th^, 90^th ^and 95^th ^percentiles of non high-density lipoprotein cholesterol for urban boys 14–18 years of age.

**Figure 6:**
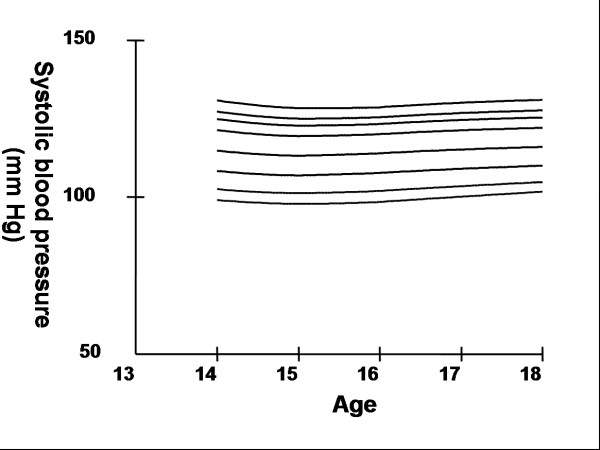
Smoothed percentile curves for the 5^th^, 10^th^, 25^th^, 50^th^, 75^th^, 85^th^, 90^th ^and 95^th ^percentiles of systolic blood pressure for urban boys 14–18 years of age.

**Figure 7:**
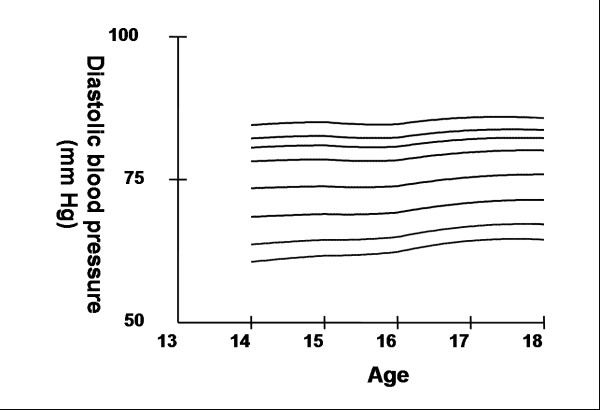
Smoothed percentile curves for the 5^th^, 10^th^, 25^th^, 50^th^, 75^th^, 85^th^, 90^th ^and 95^th ^percentiles of diastolic blood pressure for urban boys 14–18 years of age.

**Figure 8:**
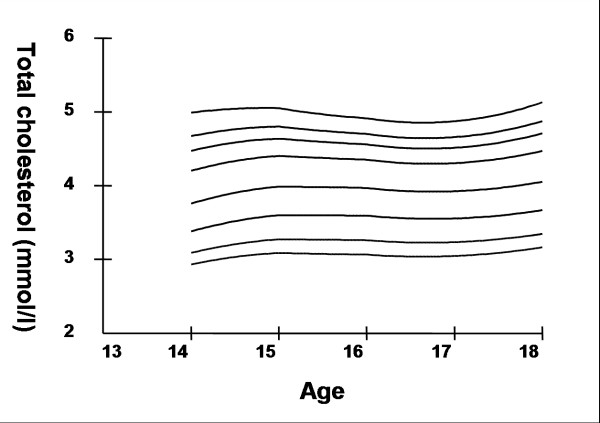
Smoothed percentile curves for the 5^th^, 10^th^, 25^th^, 50^th^, 75^th^, 85^th^, 90^th ^and 95^th ^percentiles of total cholesterol for urban girls 14–18 years of age.

**Figure 9:**
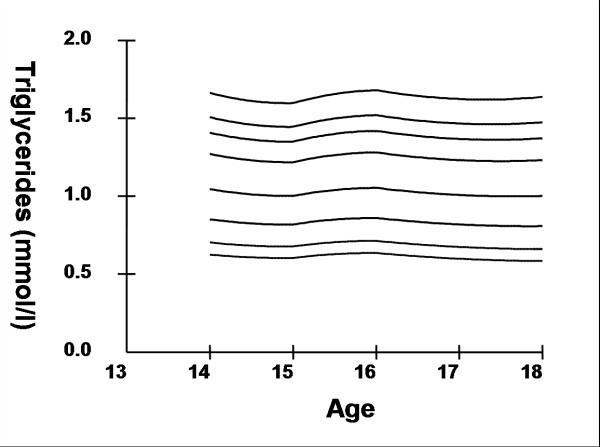
Smoothed percentile curves for the 5^th^, 10^th^, 25^th^, 50^th^, 75^th^, 85^th^, 90^th ^and 95^th ^percentiles of serum triglycerides for urban girls 14–18 years of age.

**Figure 10:**
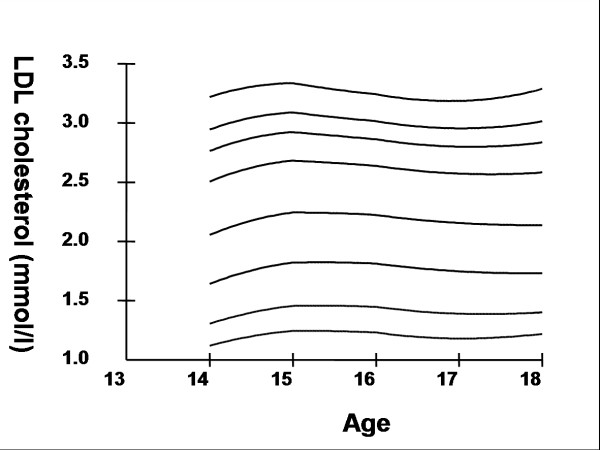
Smoothed percentile curves for the 5^th^, 10^th^, 25^th^, 50^th^, 75^th^, 85^th^, 90^th ^and 95^th ^percentiles of low-density lipoprotein cholesterol for urban girls 14–18 years of age.

**Figure 11:**
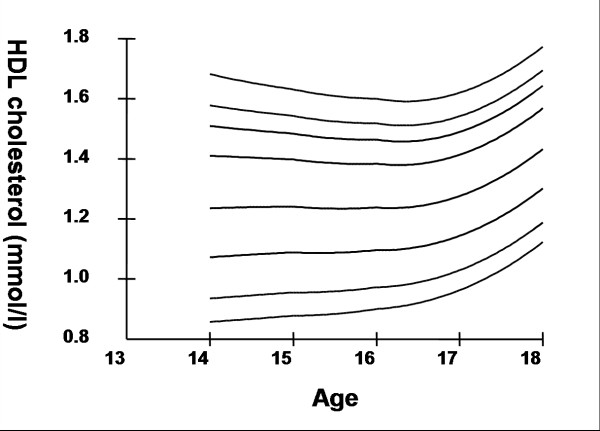
Smoothed percentile curves for the 5^th^, 10^th^, 25^th^, 50^th^, 75^th^, 85^th^, 90^th ^and 95^th ^percentiles of high-density lipoprotein cholesterol for urban girls 14–18 years of age.

**Figure 12:**
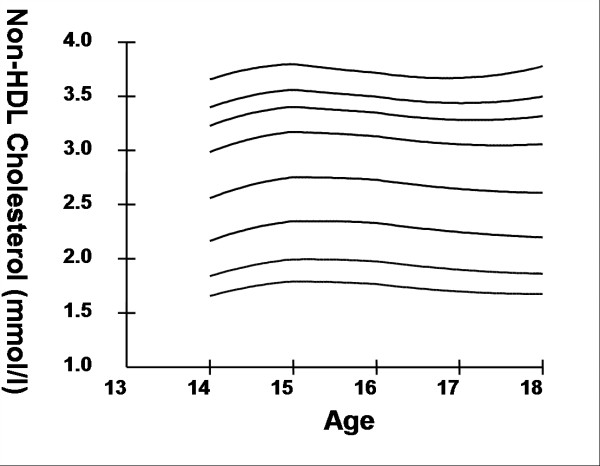
Smoothed percentile curves for the 5^th^, 10^th^, 25^th^, 50^th^, 75^th^, 85^th^, 90^th ^and 95^th ^percentiles of non high-density lipoprotein cholesterol for urban girls 14–18 years of age.

**Figure 13:**
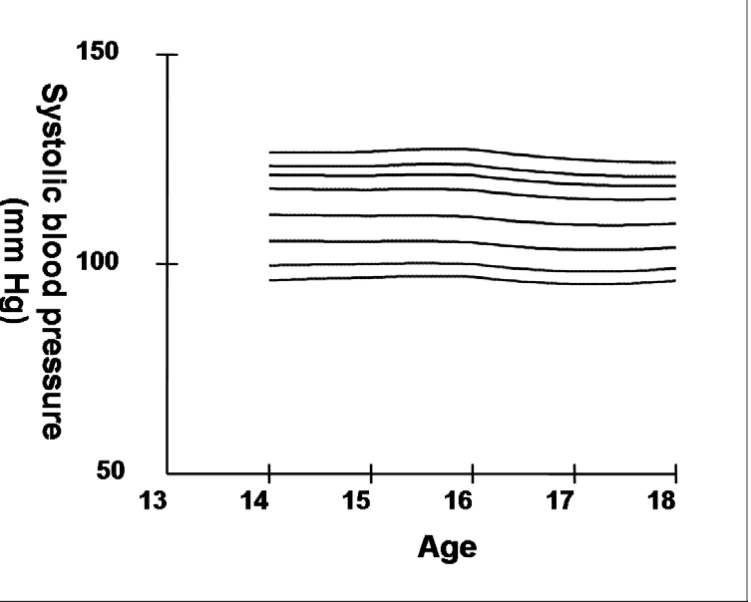
Smoothed percentile curves for the 5^th^, 10^th^, 25^th^, 50^th^, 75^th^, 85^th^, 90^th ^and 95^th ^percentiles of systolic blood pressure for urban girls 14–18 years of age.

**Figure 14:**
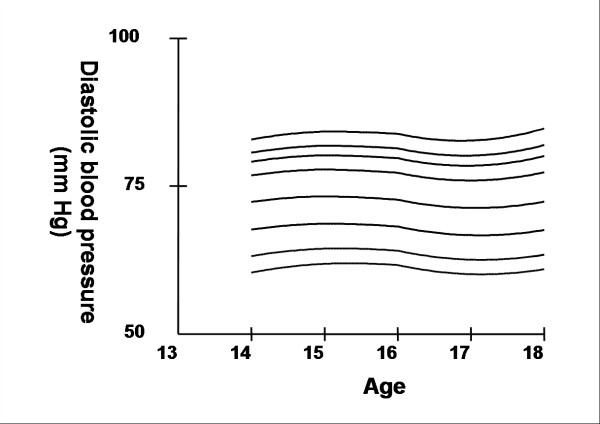
Smoothed percentile curves for the 5^th^, 10^th^, 25^th^, 50^th^, 75^th^, 85^th^, 90^th ^and 95^th ^percentiles of diastolic blood pressure for urban girls 14–18 years of age.

Boys aged 14 to 16 y showed a minimal increase in mean serum TC followed by a sharp decline at ages 17 and 18 y. Serum TC in adolescent girls, on the other hand, showed a relative minimum at 16 and17 y. Overall girls had higher mean TC than boys. The 95^th ^percentile for TC in girls aged 18 y was the highest estimate for all ages and both sexes. Mean LDL-C in both boys and girls aged 14 to18 y was highest at 15 y and decreased with age beyond 15 y. Girls had higher mean LDL-C than boys at all ages. The serum non-HDL-C levels followed a similar trend. The 95^th ^and 75^th ^percentile of TC and LDL-C (which define high and borderline elevated cholesterol respectively in children and adolescents) are of special note [18]. Mean HDL-C was relatively constant among 14 to 18 y old boys and girls except that in girls mean HDL-C levels increased 1.5 mmol/l from 17 to 18 y. The 5^th ^percentile, which would define low HDL-C in these adolescents, is also represented in table 4. Mean serum TG levels did not vary significantly among 15 to 18 year olds except for a small increase at 16 years for both sexes. The mean TG was generally higher in girls at all ages.

The mean SBP and DBP in both sexes did not vary significantly between the different age groups. Boys aged 16 to 18 y had a greater systolic and diastolic BP than girls.

## Discussion

Representative age- and sex- specific reference data on the distribution of lipids and blood pressure in urban Asian Indian adolescents aged 14 to 18 y have been provided for the first time. This data can be used as a reference for the urban Indian population for comparison to other studies, to measure the progress in health of Indian adolescents in the future, and to plan and implement intervention programs for the prevention of cardiovascular disease.

There are interesting trends in the TC and HDL-C levels in urban Indian adolescent population, which may be significantly accounted for by the marked diet and lifestyle changes in the 15 to 18 y age group. 15 to 17 y old adolescents in India are subjected to strenuous and important examinations leading to adverse diet changes and inactivity during this period [19], which may have resulted in high TC and lower HDL-C levels in these adolescents. An average urban Indian adolescent enters college at the age of 17 to 18 y, when physical activity and diet are likely to improve which could lead to lower TC and higher HDL-C levels observed in this age group.

The serum lipid levels were compared with Lipid Research Clinics (LRC) and NHANES III data from the USA [20-22]. TC and LDL-C levels were consistently lower in Asian Indians than in the USA in both sexes and across the age groups studied. Secular data in TC levels in adolescents in the USA, showed a downward trend from 1966–1970 (NHES III) to 1988–1994 (NHANES III) [22]. However, TC levels in Asian Indian adolescents were lower than those observed in the USA in the more recent NHANES III data.

The data show that TG levels in Asian Indian adolescents were lower than that observed in the NHANES III study at percentiles higher than the 75^th^, despite being higher at the lower percentiles [22]. Asian Indian adolescents had higher HDL-C levels at lower percentiles than their counterparts in the USA [22] and lower HDL-C levels at percentiles greater than the 50^th^. Although the TC and LDL-C levels are lower in urban Asian Indian adolescents than in other populations, however, the fasting hyperinsulinemia (fasting serum insulin level >20 microunits/ml)[23], indicative of insulin resistance, was seen in 50% of girls and 19% of boys, in addition to the presence of other cardiovascular risk factors such as high C-reactive protein (CRP) levels [13,14,16]. These data indicate that although lipids may be important in pathogenesis of CAD in adult Asian Indians, it may not be always possible to ascertain the risk based on lipid levels alone during childhood. The cardiovascular risk in young Asian Indians could be better estimated by assessing several other biochemical factors (insulin, CRP, non-esterified fatty acids) in addition to lipids.

## Conflict of Interest

The author(s) declare that they have no competing interests.

## References

[B1] Misra A (2000). Risk factors for atherosclerosis in young individuals. J Cardiovasc Risk.

[B2] Strong JP (1991). The natural history of atherosclerosis in childhood. Ann N Y Acad Sci.

[B3] McGill HCJ, McMahan CA, Malcom GT, Oalmann MC, Strong JP (1997). Effects of serum lipoproteins and smoking on atherosclerosis in young men and women. The PDAY Research Group. Pathobiological Determinants of Atherosclerosis in Youth. Arterioscler Thromb Vasc Biol.

[B4] McGill HCJ, McMahan CA (1998). Determinants of atherosclerosis in the young. Pathobiological Determinants of Atherosclerosis in Youth (PDAY) Research Group. Am J Cardiol.

[B5] (2001). Executive Summary of the Third Report of the National Cholesterol Education Program (NCEP) Expert Panel on Detection, Evaluation, and Treatment of High Blood Cholesterol in Adults (Adult Treatment Panel III).. JAMA.

[B6] Austin MA, Hokanson JE, Edwards KL (1998). Hypertriglyceridemia as a cardiovascular risk factor. Am J Cardiol.

[B7] Assmann G, Schulte H, Funke H, von Eckardstein A (1998). The emergence of triglycerides as a significant independent risk factor in coronary artery disease. Eur Heart J.

[B8] Grundy SM (1998). Hypertriglyceridemia, atherogenic dyslipidemia, and the metabolic syndrome. Am J Cardiol.

[B9] Gordon DJ, Probstfield JL, Garrison RJ, Neaton JD, Castelli WP, Knoke JD, Jacobs DRJ, Bangdiwala S, Tyroler HA (1989). High-density lipoprotein cholesterol and cardiovascular disease. Four prospective American studies. Circulation.

[B10] Cui Y, Blumenthal RS, Flaws JA, Whiteman MK, Langenberg P, Bachorik PS, Bush TL (2001). Non-high-density lipoprotein cholesterol level as a predictor of cardiovascular disease mortality. Arch Intern Med.

[B11] Reddy KS, Yusuf S (1998). Emerging epidemic of cardiovascular disease in developing countries. Circulation.

[B12] Misra A, Vikram NK (2004). Insulin resistance syndrome (metabolic syndrome) and obesity in Asian Indians: evidence and implications. Nutrition.

[B13] Misra A, Vikram NK, Arya S, Pandey RM, Dhingra V, Chatterjee A, Dwivedi M, Sharma R, Luthra K, Guleria R, Talwar KK (2004). High prevalence of insulin resistance in postpubertal Asian Indian children is associated with adverse truncal body fat patterning, abdominal adiposity and excess body fat. Int J Obes Relat Metab Disord.

[B14] Vikram NK, Misra A, Pandey RM, Dwivedi M, Luthra K (2004). Adiponectin levels in postpubertal asian Indian adolescents: Relationships with insulin resistance and C-reactive protein. Metabolism.

[B15] Lemeshow S, Stroh G (1988). Sampling techniques for evaluating health parameters in developing countries. National Academy Press, Washington, DC.

[B16] Vikram NK, Misra A, Dwivedi M, Sharma R, Pandey RM, Luthra K, Chatterjee A, Dhingra V, Jailkhani BL, Talwar KK, Guleria R (2003). Correlations of C-reactive protein levels with anthropometric profile, percentage of body fat and lipids in healthy adolescents and young adults in urban North India. Atherosclerosis.

[B17] Friedewald WT, Levy RI, Fredrikson DS (1972). Estimation of the concentration of low-density lipoprotein cholesterol in plasma without use of the preparative ultracentrifuge.. Clin Chem.

[B18] (1992). National Cholesterol Education Program (NCEP): highlights of the report of the Expert Panel on Blood Cholesterol Levels in Children and Adolescents. Pediatrics.

[B19] Dhingra V, Chatterjee A, Guleria R, Sharma R, Pandey RM, Talwar KK, Misra A (2002). Adverse physical activity pattern in urban adolescents. J Assoc Physicians India.

[B20] (1992). American Academy of Pediatrics. National Cholesterol Education Program: Report of the Expert Panel on Blood Cholesterol Levels in Children and Adolescents. Pediatrics.

[B21] (1979). Plasma lipid distributions in selected North American populations: the Lipid Research Clinics Program Prevalence Study. The Lipid Research Clinics Program Epidemiology Committee. Circulation.

[B22] Hickman TB, Briefel RR, Carroll MD, Rifkind BM, Cleeman JI, Maurer KR, Johnson CL (1998). Distributions and trends of serum lipid levels among United States children and adolescents ages 4-19 years: data from the Third National Health and Nutrition Examination Survey. Prev Med.

[B23] Alberti KG, Zimmet PZ (1998). Definition, diagnosis and classification of diabetes mellitus and its complications. Part 1: diagnosis and classification of diabetes mellitus provisional report of a WHO consultation. Diabet Med.

